# Association of Work Requirements With Supplemental Nutrition Assistance Program Participation by Race/Ethnicity and Disability Status, 2013-2017

**DOI:** 10.1001/jamanetworkopen.2020.5824

**Published:** 2020-06-26

**Authors:** Erin Brantley, Drishti Pillai, Leighton Ku

**Affiliations:** 1 Milken Institute School of Public Health at the George Washington University, Washington, District of Columbia

## Abstract

**Importance:**

Increased work requirements have been proposed throughout federal safety net programs, including the Supplemental Nutrition Assistance Program (SNAP). Participation in SNAP is associated with reduced food insecurity and improved health.

**Objectives:**

To determine whether SNAP work requirements are associated with lower rates of program participation and to examine whether there are racial/ethnic disparities or spillover effects for people with disabilities, who are not intended to be affected by work requirements.

**Design, Setting, and Participants:**

This nationally representative, pooled cross-sectional study examined how changes in SNAP work requirements at state and local levels in the US are associated with changes in food voucher program participation. The study combined information on state and local SNAP work requirements with repeated cross-sections from the 2012 through 2017 American Community Survey (with outcomes covering 2013 to 2017). The analytical approaches were based on difference-in-difference and triple-difference methods, after controlling for other economic and social factors. The sample included low-income adults without dependents, stratified by racial/ethnic group and disability status. The study also included parents who would otherwise meet work requirement criteria as a comparison group to estimate triple-difference models. This accounted for otherwise unobserved factors affecting trends in SNAP participation within local areas. Data were analyzed from January 2019 through March 2020.

**Exposure:**

Residence in areas where SNAP work requirements apply.

**Main Outcomes and Measures:**

The primary outcome is SNAP participation measured by whether anyone in the household received food vouchers at any point over the prior 12 months.

**Results:**

The final analytical sample included 866 000 low-income adults (weighted mean [SE] age, 33.6 [0.01] years; 42.5% [SE, 0.07%] men). The racial/ethnic breakdown was 56.5% (SE, 0.07%) non-Hispanic white respondents, 19.4% (SE, 0.06%) non-Hispanic black respondents, 17.7% (SE, 0.06%) Hispanic respondents, 2.5% (SE, 0.02%) Asian respondents, and 3.9% (SE, 0.03%) respondents of other or multiple races. In final triple-difference models, work requirements were associated with a 4.0 percentage point decrease in participation (95% CI, –0.048 to –0.032; *P* < .001) for childless adults without disability, equivalent to a 21.2% reduction in SNAP participation (95% CI, –25.5% to –17.0%). For childless adults with disability, work requirements were associated with a 4.0 percentage point reduction (95% CI, –0.058 to –0.023; *P* < .001), equivalent to 7.8% fewer SNAP participants with disability (95% CI, –11.2% to –4.4%). When the final models were stratified by race/ethnicity, benefit reductions were larger for non-Hispanic black adults (7.2 percentage points; 95% CI, –0.092 to –0.051; *P* < .001) and Hispanic adults (5.5 percentage points; 95% CI, –0.072 to –0.038; *P* < .001) than for non-Hispanic white adults (2.6 percentage points; 95% CI, –0.035 to –0.016; *P* < .001).

**Conclusions and Relevance:**

Because of the association of SNAP with food security and health, work requirements that lead to benefit loss may create nutritional and health harm for low-income Americans. These findings suggest that there may be racially disparate consequences and unintended harm for those with disability.

## Key Points

**Question:**

What are the associations between work requirements and Supplemental Nutrition Assistance Program participation for the overall target population and subpopulations?

**Findings:**

In this pooled cross-sectional study of 866 000 low-income US adults, work requirements were associated with a 4.0 percentage point decrease in participation for the target population of childless adults without disability, with reductions in participation of 7.2 percentage points for non-Hispanic black adults, 5.5 percentage points for Hispanic adults, and 2.6 percentage points for non-Hispanic white adults within this group. There was also a 4.0 percentage point decrease for childless adults with disability.

**Meaning:**

These findings suggest that Supplemental Nutrition Assistance Program work requirements are associated with decreased participation for subpopulations who have greater rates of food insecurity.

## Teaser

This cross-sectional study examines whether work requirements in the Supplemental Nutrition Assistance Program (SNAP) are associated with lower program participation rates and whether there are racial/ethnic disparities or spillover effects for people with disabilities.

## Introduction

The Supplemental Nutrition Assistance Program (SNAP) is a major entitlement benefit, providing 37 million people with food assistance as of January 2020.^1^ Given the economic crisis associated with the coronavirus disease 2019 pandemic, SNAP participation will likely increase further. Low-income people who receive SNAP benefits, which are electronic vouchers to help purchase groceries, have improved food security^2^ and self-assessed health.^3^ Receiving SNAP benefits is associated with reduced health care utilization^4^ and expenditures.^5,6^

Since 1997, SNAP participants categorized as ABAWD (able-bodied adults without dependents, referred to hereafter as adults without disability) aged 18 to 49 years have been limited to 3 months of SNAP benefits in any 36-month period unless they work 80 or more hours per month or participate in an approved work training program.^7^ (Some other SNAP recipients are subject to much less stringent requirements to accept a suitable job offer, and so forth.)^7^ When unemployment rates are high, states may apply to waive work requirements. During the Great Recession, work requirements were waived throughout most of the country. Work requirements were reinstated after 2013, with the largest increase occurring in 2016 (Figure). The Families First Coronavirus Response Act paused SNAP work requirements until the coronavirus disease 2019 public health emergency declared by the Secretary of Health and Human Services is lifted.^8^

**Figure.**
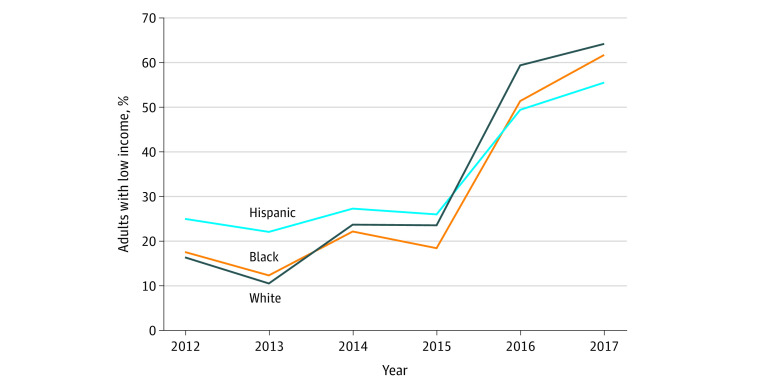
Percentage of Low-income Adults Living in Work Requirement Areas, 2012-2017

Multiple studies have reported that SNAP work requirements lead to substantial decreases in participation,^9,10,11,12^ but little is known about the characteristics of people who lose benefits. This is despite uneven labor market outcomes by race/ethnicity^13^ and prior research indicating that racial/ethnic minority cash assistance recipients were more likely than non-Hispanic white recipients to face penalties associated with work requirements.^14^ One study found similar outcomes associated with work requirements for SNAP participation of non-Hispanic white and non-Hispanic black adults, but the association was significant only for non-Hispanic white adults.^11^

To our knowledge, no prior studies have examined whether SNAP work requirements for adults without disability have spillover effects on people with disabilities. People receiving government disability benefits such as Supplemental Security Income (SSI) are automatically exempt from SNAP work requirements,^15^ and others with disabilities can seek an exemption. However, adults with disability may not understand how to pursue or may not be granted an exemption.

This study seeks to expand our understanding of distributional effects of SNAP work requirements intended to apply to adults without disability. We pose the following research questions: Does the impact of SNAP work requirements vary by race/ethnicity? Do SNAP work requirements have spillover effects on adults with disabilities?

## Methods

In with accordance George Washington University’s institutional review board policy, this study was determined to be exempt from review because we used publicly available deidentified data. Therefore, waiver of informed consent did not apply. This cross-sectional study follows the Strengthening the Reporting of Observational Studies in Epidemiology (STROBE) reporting guideline.

### Data

We used 2012 to 2017 public-use versions of the American Community Survey (ACS) provided by the US Census Bureau and obtained information on each individual’s parental status from Integrated Public Use Microdata Series USA.^16^ Because we use lagged independent variables, our outcomes cover 2013 to 2017. There are no missing data due to US Census Bureau imputation for item nonresponse.^17^

The Food and Nutrition Service provided quarterly information on waivers of SNAP work requirements at the municipality, county, and state levels from 2012 through 2017. Our analyses were conducted at the Public Use Micro Area (hereafter called area) level, the smallest geographic unit publicly available in the ACS. These 2351 areas are geographically contiguous, nested within states, and contain approximately 100 000 or more people.^18^ To translate local work requirement data to the area level, we used Missouri Population Center data to estimate the percentage of residents of each area who lived in a place where the work requirement was in effect.^19^

### Study Sample

Our sample includes individuals with household incomes below 200% of the federal poverty level because higher-income people are unlikely to be affected by SNAP policy. We stratified by disability status. In alignment with federal regulations,^20^ our sample of adults without disability excludes those who would be exempt from work requirements for able-bodied adults without dependents: children and people living in a household with children, adults older than 49 years, pregnant women (proxied by having given birth within the past 12 months), and people with a disability (as indicated by receiving SSI or reporting ≥1 of 6 functional limitations). In addition, we excluded groups that face other specific SNAP eligibility policies: noncitizens, current students, people living in households with elderly members (aged ≥60 years) or members officially with disability (receiving SSI), and people living in group quarters. The sample with disability comprises people who would meet these same criteria but report having a functional limitation, including people who do and do not receive SSI.^21^

### Treatment and Outcome Measures

Our outcome, SNAP participation, is based on responses to whether anyone in the household received food vouchers at any point over the prior 12 months. The ACS is fielded throughout the year. The interview date is not provided. To address this wide time frame, our treatment variable is a weighted estimate of the proportion of each area covered by work requirements over the survey year and the prior year (see eAppendix in the Supplement).

### Covariates

In addition to race/ethnicity, individual covariates include gender, educational attainment, marital status, home ownership, household size, and age. We used ACS data to estimate the unemployment rate in each area in the current and prior year and the poverty rate in the current year. We controlled for Medicaid eligibility level as reported by the Kaiser Family Foundation for childless adults and for parents (in appropriate models) because prior research has associated Medicaid expansion with SNAP participation.^22,23,24^ Medicaid eligibility was coded as the ratio of the eligibility level to the federal poverty line in each state and year.

### Statistical Analysis

We estimated difference-in-difference and difference-in-difference-in-difference (triple-difference) models.^25^ In the basic models, we analyzed changes across years in areas with and without work requirements, using linear probability models for ease of interpretation. Area fixed effects controlled for time-invariant factors (eg, rurality or types of employment). Year fixed effects controlled for national-level trends, such as temporary benefit level changes between 2009 and 2013.^26^ In addition to results for the full sample without disabilities, we estimated associations for stratified samples of non-Hispanic white, non-Hispanic black, and Hispanic adults without disabilities. See the eAppendix in the Supplement for more details on analytical models.

To address the possibility that unobserved factors could be associated with when work requirements were applied in different areas, we refined our analyses by estimating triple-difference models comparing effects across a third dimension that differentiates the policy but shares the influence of any unobserved factors associated with participation. Our main triple-difference models compared childless adults vs parents, who should not be affected by work requirements. If there were unobserved changes in local economic or social factors that modified SNAP participation, they should have applied similarly to childless adults and parents. Prior working papers^10,11,12^ have used the able-bodied adults without dependents age cutoff (age ≤49 years vs age >49 years) to construct triple-difference models. Our use of parents as the comparison group permitted analysis of the entire age range, which has greater policy applicability and provides larger sample sizes.

We addressed the ACS’s complex survey design by using ACS survey weights and clustering by area. Analyses were conducted using Stata MP statistical software version 13 (StataCorp) from January 2019 through March 2020. We assumed statistical significance at the 2-tailed *P* < .05 level.

### Robustness Checks

We conducted 2 robustness tests. As an alternative to using parents as a comparison group, we ran triple-difference models comparing participation for adults aged 45 to 49 years vs those aged 50 to 54 years, similar to prior analyses.^10,11,12^ For the sample with disability, we ran versions of our models excluding individuals who receive SSI, who are automatically exempt.

## Results

The final analytical sample included 866 000 low-income adults (weighted mean [SE] age, 33.6 [0.01] years; 42.5% [SE, 0.07%] men). The racial/ethnic breakdown was 56.5% (SE, 0.07%) non-Hispanic white respondents, 19.4% (SE, 0.06%) non-Hispanic black respondents, 17.7% (SE, 0.06%) Hispanic respondents, 2.5% (SE, 0.02%) Asian respondents, and 3.9% (SE, 0.03%) respondents of other or multiple races. Additional characteristics are described in eTable 1 in the Supplement.

Our sample included 272 393 childless adults without disabilities. The weighted mean (SE) age was 31.37 (0.02) years, and 52.66% (SE, 0.12%) were men. The percentages for race/ethnicity were 59.71% (SE, 0.12%) non-Hispanic white respondents, 19.41% (SE, 0.10%) non-Hispanic black respondents, 14.37% (SE, 0.09%) Hispanic respondents, 2.72% (SE, 0.04%) Asian respondents, and 3.79% (SE, 0.05%) respondents of other or multiple races (Table 1). The sample of 71 148 childless adults with disabilities had a weighted mean (SE) age of 36.92 (0.05) years (52.58% [SE, 0.24%] male). The percentages for race/ethnicity were 64.14% (SE, 0.24%) non-Hispanic white respondents, 19.07% (SE, 0.20%) non-Hispanic black respondents, 10.84% (SE, 0.16%) Hispanic respondents, 0.94% (SE, 0.04%) Asian respondents, and 5.01% (SE, 0.11%) respondents of other or multiple races. Our comparison groups included 462 905 parents without disability and 59 550 parents with disability. The 2 groups of parents were similar in terms of race/ethnicity (percentages for those without disability vs those with disability, non-Hispanic white, 52.82% [SE, 0.10%] vs 58.98% [SE, 0.27%]; non-Hispanic black, 19.46% [SE, 0.08%] vs 19.12% [SE, 0.22%]), education (percentage with a high school education, 37.29% [0.09%] for those without disability vs 36.08% [0.26%] for those with disability), and age (mean [SE] age, 34.11 [0.01] years for those without disability vs 36.50 [0.04] years for those with disability). Compared with childless adults, parents were more likely to be female (percentage male, 52.66% [SE, 0.12%] of childless adults without disability and 52.58% [SE, 0.24%] childless adults with disability vs 35.33% [SE, 0.09%] of parents without disability and 34.42% [SE, 0.25%] of parents with disability) and married (percentage married, 13.04% [SE, 0.08%] of childless adults without disability and 14.24% [SE, 0.16%] of childless adults with disability vs 50.62% [SE, 0.10%] of parents without disability and 42.69% [SE, 0.26%] of parents with disability).

**Table 1.** Characteristics of Low-income Adults With and Without Disabilities and Comparison Groups

Characteristic	Weighted % (SE)^a^			
	Able-bodied adults without dependents (n = 272 397)	Adults with disability (n = 71 148)	Parents without disability (n = 462 905)	Parents with disability (n = 59 550)
Race/ethnicity				
Non-Hispanic				
White	59.71 (0.12)	64.14 (0.24)	52.82 (0.10)	58.98 (0.27)
Black	19.41 (0.10)	19.07 (0.20)	19.46 (0.08)	19.12 (0.22)
Asian	2.72 (0.04)	0.94 (0.04)	2.84 (0.03)	1.19 (0.06)
Hispanic	14.37 (0.09)	10.84 (0.16)	21.26 (0.08)	15.67 (0.20)
Other or multiple races	3.79 (0.05)	5.01 (0.11)	3.62 (0.04)	5.04 (0.12)
Male	52.66 (0.12)	52.58 (0.24)	35.33 (0.09)	34.42 (0.25)
Education				
Less than high school	11.95 (0.08)	24.07 (0.21)	15.80 (0.07)	23.94 (0.23)
High school	37.87 (0.12)	40.57 (0.24)	37.29 (0.09)	36.08 (0.26)
Some college	31.99 (0.12)	28.02 (0.22)	35.62 (0.09)	34.03 (0.25)
College graduate	18.19 (0.09)	7.34 (0.12)	11.29 (0.06)	5.95 (0.12)
Married	13.04 (0.08)	14.24 (0.16)	50.62 (0.10)	42.69 (0.26)
Rents	71.89 (0.11)	71.40 (0.21)	62.03 (0.09)	67.19 (0.25)
Household size, mean (SE), No. of members	2.14 (0.00)	1.96 (0.00)	4.25 (0.00)	4.07 (0.01)
Age, mean (SE), y	31.37 (0.02)	36.92 (0.05)	34.11 (0.01)	36.50 (0.04)
Medicaid eligibility, percentage of federal poverty level	63.72 (0.17)^b^	64.10 (0.33)^b^	89.64 (0.10)^c^	91.15 (0.28)^c^
Unemployment rate	7.23 (0.01)	7.51 (0.02)	7.35 (0.01)	7.60 (0.02)
Poverty rate	16.97 (0.02)	17.65 (0.04)	16.86 (0.01)	17.58 (0.04)

^a^ Weighted estimates of 2013-2017 American Community Survey data.

^b^ Medicaid eligibility level for adults without dependents.

^c^ Medicaid eligibility level for parents.

Table 2 presents initial difference-in-difference findings. The presence of work requirements was associated with a 3.5 percentage point decrease in reported SNAP participation among low-income adults without disability (95% CI, –0.045 to –0.026; *P* < .001). The coefficient reflects the change from no work requirement in an area over the 2-year window to complete coverage with a work requirement. Given that 19% of low-income adults without disability participate in SNAP, a 3.5 percentage point reduction is equivalent to an 18.9% relative reduction in the number of SNAP participants (95% CI, –24.1% to –13.7%). Consistent with prior research, SNAP participation is also associated with current (0.5 percentage point increase; 95% CI, 0.003 to 0.006; *P* < .001) and prior year (0.1 percentage point increase; 95% CI, 0.000 to 0.003; *P* = .06) unemployment, the current poverty rate (0.2 percentage point increase; 95% CI, 0.001 to 0.003; *P* < .001), and Medicaid eligibility (1.1 percentage point increase; 95% CI, 0.003 to 0.018; *P* = .004).

**Table 2.** Associations of Work Requirements and Supplemental Nutrition Assistance Program Participation, Difference-in-Difference Models^a^

Variable	Able-bodied adults without dependents, percentage point change (95% CI)				Adults with disability (n = 71 148), percentage point change (95% CI)^b^
	All (N = 272 397)^b^	Non-Hispanic black (n = 41 269)	Hispanic (n = 35 163)	Non-Hispanic white (n = 176 140)	
Work requirement	–0.035 (–0.045 to –0.026)	–0.068 (–0.097 to –0.039)	–0.019 (–0.047 to 0.009)	–0.025 (–0.036 to –0.014)	–0.044 (–0.066 to –0.022)
Unemployment rate	0.005 (0.003 to 0.006)	0.005 (0.001 to 0.009)	0.005 (0.001 to 0.010)	0.004 (0.002 to 0.006)	0.004 (0.001 to 0.007)
Unemployment rate, 1 y earlier	0.001 (0.000 to 0.003)	0 (–0.003 to 0.004)	0.001 (–0.003 to 0.004)	0.001 (–0.001 to 0.003)	0.002 (–0.001 to 0.005)
Poverty rate	0.002 (0.001 to 0.003)	0.003 (0.000 to 0.006)	0.002 (0.000 to 0.005)	0.002 (0.000 to 0.003)	0.002 (0 to 0.004)
Adult Medicaid eligibility	0.011 (0.003 to 0.018)	–0.003 (–0.026 to 0.020)	0.013 (–0.007 to 0.033)	0.014 (0.005 to 0.022)	0.023 (0.006 to 0.039)
Supplemental Nutrition Assistance Program participation, mean, %	0.19	0.31	0.18	0.15	0.52
Implied percentage reduction	–18.9 (–24.1 to –13.7)	–21.9 (–31.3 to –12.5)	–10.5 (–26.3 to 5.3)	–16.3 (–23.3 to –9.3)	–8.5 (–12.7 to –4.2)

^a^ Data are from our analysis of 2012 to 2017 American Community Survey data using linear probability models with area and year fixed effects. Models also control for age, gender, marital status, education, household size, and home ownership.

^b^ Model also controls for race/ethnicity.

Models stratified by race/ethnicity indicate disparate associations of work requirements and SNAP participation. Table 2 shows a 6.8 percentage point reduction for non-Hispanic black childless adults without disabilities (95% CI, –0.097 to –0.039; *P* < .001), compared with a 2.5 percentage point (95% CI, –0.036 to –0.014; *P* < .001) reduction for non-Hispanic white adults without disability; these are equivalent to 21.9% (95% CI, –31.3% to –12.5%) and 16.3% (95% CI, –23.3% to –9.3%) reductions in the number of SNAP participants, respectively. For adults with disability, there is a 4.4 percentage point reduction (95% CI, –0.066 to –0.022; *P* < .001). Because approximately 50% of those with disabilities participate in SNAP, this is equivalent to an 8.5% relative reduction in SNAP enrollment (95% CI, –12.7% to –4.2%). eTable 2 in the Supplement shows results with covariates.

Final triple-difference models including parents as a comparison group are shown in Table 3 (also see eTable 3 in the Supplement). The coefficient for the interaction of work requirements and able-bodied adults without dependents status indicates the association of work requirements with SNAP participation. Results are similar to those shown in Table 2. There is a 4.0 percentage point decrease (95% CI, –0.048 to –0.032; *P* < .001) for childless adults compared with parents, equivalent to a 21.2% decrease in SNAP enrollment (95% CI, –25.5% to –17.0%). The associations of work requirements and SNAP participation appear to be larger for non-Hispanic black (decrease of 7.2 percentage points; 95% CI, –0.092 to –0.051; *P* < .001) and Hispanic (decrease of 5.5 percentage points; 95% CI, –0.072 to –0.038; *P* < .001) adults than for non-Hispanic white adults (decrease of 2.6 percentage points; 95% CI, –0.035 to –0.016; *P* < .001). There are significant associations for childless adults with disability, including an overall 4.0 percentage point reduction (95% CI, –0.058 to –0.023; *P* < .001) in participation. Work requirements are associated with a 23.1% relative reduction in SNAP enrollment for non-Hispanic black adults (95% CI, –29.8% to –16.4%), a 30.9% reduction for Hispanic adults (95% CI, –40.6% to –21.3%), and a 16.4% reduction for non-Hispanic white adults (95% CI, –22.6% to –10.3%). Those with disabilities experience a 7.8% relative decline in SNAP participation (95% CI, –11.2% to –4.4%).

**Table 3.** Associations of Work Requirements and Supplemental Nutrition Assistance Program Participation, Triple-Difference Models Comparing Childless Adults vs Parents^a^

Variable	Able-bodied adults without dependents, percentage point change (95% CI)				Adults with disability (n = 130 698), percentage point change (95% CI)^b^
	All (N = 735 302)^b^	Non-Hispanic black (n = 109 330)	Hispanic (n = 121 189)	Non-Hispanic white (n = 451 503)	
Work requirement	–0.006 (–0.014 to 0.002)	–0.002 (–0.021 to 0.016)	–0.005 (–0.024 to 0.013)	–0.006 (–0.016 to 0.004)	–0.009 (–0.027 to 0.010)
Able-bodied adult without dependent (vs parent)	–0.178 (–0.184 to –0.172)	–0.175 (–0.189 to –0.162)	–0.181 (–0.194 to –0.168)	–0.171 (–0.179 to –0.164)	–0.080 (–0.092 to –0.069)
Work requirement and able-bodied adult without dependent interaction	–0.040 (–0.048 to –0.032)	–0.072 (–0.092 to –0.051)	–0.055 (–0.072 to –0.038)	–0.026 (–0.035 to –0.016)	–0.040 (–0.058 to –0.023)
Unemployment rate	0.004 (0.003 to 0.005)	0.004 (0.002 to 0.006)	0.002 (–0.001 to 0.004)	0.005 (0.003 to 0.006)	0.003 (0.001 to 0.005)
Unemployment rate, 1 y earlier	0.001 (0 to 0.002)	0 (–0.002 to 0.002)	0.001 (–0.001 to 0.003)	0.002 (0 to 0.003)	0 (–0.002 to 0.003)
Poverty rate	0.004 (0.003 to 0.004)	0.003 (0.001 to 0.005)	0.004 (0.002 to 0.005)	0.004 (0.003 to 0.005)	0.004 (0.003 to 0.006)
Adults Medicaid eligibility	0.010 (0.003 to 0.017)	–0.003 (–0.022 to 0.015)	0.016 (–0.001 to 0.034)	0.010 (0.001 to 0.018)	0.016 (–0.001 to 0.032)
Parental Medicaid eligibility	–0.010 (–0.020 to 0.001)	0.004 (–0.024 to 0.033)	–0.002 (–0.035 to 0.032)	–0.013 (–0.025 to –0.001)	–0.014 (–0.036 to 0.008)
Supplemental Nutrition Assistance Program participation, mean, %	0.19	0.31	0.18	0.15	0.52
Implied percentage reduction	–21.2 (–25.5 to –17.0)	–23.1 (–29.8 to –16.4)	–30.9 (–40.6 to –21.3)	–16.4 (–22.6 to –10.3)	–7.8 (–11.2 to –4.4)

^a^ Data are from our analysis of 2012 to 2017 American Community Survey data using linear probability models with area and year fixed effects. Models also control for age, gender, marital status, education, household size, and home ownership.

^b^ Model also controls for race/ethnicity.

Table 4 presents results for an alternative triple-difference specification comparing adults around the age 49-year cutoff (45-49 vs 50-54 years old) (see also eTable 4 in the Supplement). For the interaction between work requirements and age 45 to 49 years, we found associations for adults without disabilities overall (2.1 percentage point decrease; 95% CI, –0.036 to –0.007; *P* = .005), non-Hispanic black adults without disabilities (5.9 percentage point decrease; 95% CI, –0.101 to –0.018; *P* = .005), and adults with disability (4.0 percentage point decrease; 95% CI, –0.065 to –0.016; *P* = .001), consistent with previous findings. However, no significant associations for non-Hispanic white (1.2 percentage point decrease; 95% CI, –0.030 to 0.005) or Hispanic (0.3 percentage point decrease; 95% CI, –0.045 to 0.039) adults without disability were found in this version, suggesting differences between younger and older non-Hispanic white and Hispanic adults.

**Table 4.** Associations of Work Requirements and Supplemental Nutrition Assistance Program Participation, Triple-Difference Models Comparing Adults Aged 45 to 49 Years vs Those Aged 50 to 54 Years^a^

Variable	Able-bodied adults without dependents, percentage point change (95% CI)				Adults with disability, percentage point change (95% CI) (n = 65 847)^b^
	All (N = 109 933)^b^	Non-Hispanic black (n = 18 454)	Hispanic (n = 11 158)	Non-Hispanic white (n = 72 981)	
Work requirement	–0.026 (–0.041 to –0.010)	–0.031 (–0.076 to 0.014)	–0.02 (–0.078 to 0.038)	–0.029 (–0.049 to –0.010)	0.007 (–0.018 to 0.033)
Work requirement and age 45-49 y interaction	–0.021 (–0.036 to –0.007)	–0.059 (–0.101 to –0.018)	–0.003 (–0.045 to 0.039)	–0.012 (–0.030 to 0.005)	–0.04 (–0.065 to –0.016)
Unemployment rate	0.003 (0.001 to 0.005)	0 (–0.004 to 0.005)	0.001 (–0.007 to 0.008)	0.004 (0.001 to 0.007)	–0.001 (–0.004 to 0.003)
Unemployment rate, 1 y earlier	0.002 (0.000 to 0.004)	0.004 (–0.000 to 0.008)	0 (–0.007 to 0.006)	0.001 (–0.001 to 0.004)	0.001 (–0.002 to 0.004)
Poverty rate	0.002 (0.000 to 0.003)	0.006 (0.002 to 0.009)	0.001 (–0.004 to 0.006)	0.001 (–0.001 to 0.002)	0.002 (–0.001 to 0.004)
Adult Medicaid eligibility	0.016 (0.005 to 0.026)	–0.017 (–0.047 to 0.014)	0.024 (–0.014 to 0.063)	0.024 (0.011 to 0.036)	0.035 (0.019 to 0.052)
Supplemental Nutrition Assistance Program participation, mean, %	0.19	0.31	0.18	0.15	0.52
Implied percentage reduction	–11.2 (–19.1 to –3.5)	–19.1 (–32.6 to –5.7)	–1.7 (–25.6 to 22.3)	–7.7 (–19.3 to 3.3)	–7.7 (–12.5 to –3.1)

^a^ Data are from our analysis of 2012 to 2017 American Community Survey data using linear probability models with area and year fixed effects. Models also control for age, gender, marital status, education, household size, and home ownership.

^b^ Model also controls for race/ethnicity.

To refine models related to disabilities, we excluded adults with disability who receive SSI, who are automatically exempt from work requirements. This yielded a significant and somewhat higher estimate of the association (see eTable 5 in the Supplement for full results).

## Discussion

The findings of this study suggest that work requirements substantially reduce SNAP benefits for childless adults, consistent with earlier analyses.^9,10,11,12^ We also identified negative associations for adults with disability and racially disparate associations for adults without disability, both new contributions. Our findings are generally consistent across alternative estimation strategies.

We improved on prior studies by comparing responses of childless adults vs parents. Three similar studies^10,11,12^ identified associations by comparing those older and younger than the age 49-year cutoff. This approach may underestimate the true association if adults who lose benefits because of work requirements are unaware that they could regain benefits when they reach age 50 years.

Prior research^10,11,12^ has found that SNAP work requirements for able-bodied adults without dependents have little to no impact on labor outcomes. Similarly, a systematic review^27^ found that introducing work requirements to welfare had, at best, modest, short-term associations with income and did not improve health. Evidence indicates that Arkansas’ Medicaid work requirement, implemented in 2018, did not improve employment.^28^

The result for adults with disability is troubling because SNAP work requirements are intended to encourage work among those without disabilities. The effect could be associated with a combination of factors. People may have a disability yet not qualify for an exemption. Alternatively, people may not complete the exemption process because of paperwork barriers. The effect became stronger when we excluded people who receive SSI, indicating that the work requirement has less or no effect among people who are automatically exempt, which is consistent with prior evidence that take-up of public programs is heavily influenced by the complexity of the application process.^29,30,31^

Non-Hispanic black adults appear to experience greater SNAP losses than non-Hispanic white adults, raising concerns about racial/ethnic disparities. Non-Hispanic black workers typically have higher unemployment rates than any other major racial/ethnic group.^13^ After the Great Recession, unemployment rates decreased sooner for non-Hispanic white workers than their non-Hispanic black counterparts.^32^ Field experiments show that non-Hispanic black job applicants often experience discrimination in hiring, and a recent meta-analysis^33^ found that this bias has not declined in the past quarter century.

This challenging labor environment may make it more difficult for racial/ethnic minorities to meet work requirements, something that SNAP work requirement policies do not consider. States can choose to seek exemptions for high unemployment in specific geographic areas, but this does not account for variations in unemployment by race/ethnicity within areas. Besides work, the only qualifying activity to meet the SNAP work requirement is participating in an approved training program. States are not required to ensure that there is sufficient capacity for all SNAP recipients who want to enroll in training.^7^ Job search activities do not qualify.

Extensive evidence from Temporary Assistance for Needy Families suggests that case worker biases can contribute to differences in the application of work requirements across race/ethnicity. Some studies^14,34,35,36^ have found that case workers apply sanctions more often for racial/ethnic minorities. We are unaware of research assessing whether similar patterns hold for SNAP work requirements.

Using ACS data produces nationally representative results; however, many important questions are left for future research. We are unable to determine whether the greater losses for black non-Hispanic adults are predominantly associated with differences in unemployment rates by race/ethnicity, differential drop-off among people who are meeting or exempt from the requirement, or some other factor. In-depth participant surveys, experiments to study caseworker responses in association with race/ethnicity, or administrative data analyses could explore underlying causes of the difference observed here. Similarly, survey or qualitative research could show how and why people with disabilities lose benefits.

The US federal government has sought to greatly expand work requirements in SNAP and other public benefit programs.^37,38^ The FNS has finalized a regulation to reduce states’ ability to waive SNAP work requirements, which it estimated will cause 755 000 adults to lose benefits.^39^ The Centers for Medicare & Medicaid Services has encouraged states to submit demonstration projects imposing work requirements in Medicaid.^38^ Litigation has thus far prevented implementation of the SNAP policy change^40^ and, for many states, the Medicaid change.^41^ The Families First Coronavirus Response Act suspended SNAP work requirements and paused most disenrollment from Medicaid, but these temporary changes are tied to the public health emergency.^8^ High unemployment could persist after the public health emergency ends, and our findings indicate that restarting work requirement policies could have disparate effects on socioeconomically vulnerable populations.

Access to adequate and healthy food is recognized as a core social determinant of health.^42^ A recent survey^43^ found that most states require Medicaid managed care companies to screen enrollees for social needs. Our findings underscore that reinstating and widening SNAP work requirements could undercut such efforts by decreasing access to food, including for adults with disabilities, who are more likely to experience food insecurity.^44^ Harm would be compounded if individuals lost access to both Medicaid and food assistance.

### Limitations

This study has several limitations. The ACS asked respondents to report any food voucher use in the prior 12 months; we cannot distinguish households that received SNAP for a limited time vs the whole year. Because the ACS is fielded continuously and the SNAP question has a 12-month look-back period, we could not assess the specific timing of the association between work requirements and participation. In addition, an estimated 35% of participating households do not report receiving SNAP in the ACS survey.^45^ Given the overall consistency of our results using alternate comparison groups, the presence or absence of work requirements is unlikely to be associated with underreporting.

These limitations suggest that our results may underestimate true outcomes. Another recent study^9^ using administrative data that measured monthly SNAP participation in counties across the US estimated that work requirements reduced SNAP participation by more than one-third.

Because of insufficient data, we did not include other SNAP policies that could affect participation. States can, at their discretion, exempt up to 15% of adults without disabilities from work requirements.^7^ Omitting this variable may have attenuated our findings. Other SNAP policies associated with participation in prior research, particularly Broad-Based Categorical Eligibility and short recertification periods,^46,47^ changed little over the study period. From 2013 through 2016, only 1 state adopted or rescinded Broad-Based Categorical Eligibility, and states consistently applied short certification periods to less than 6% of participants.^48^

We use nonexperimental data and thus cannot conclude that the association between work requirements and SNAP participation is causal. However, we used rigorous difference-in-difference and triple-difference methods, which are considered strong analytical strategies.^25^ Multiple alternative specifications supported our main findings, which are compatible with other research about work requirements showing harmful outcomes and almost no positive outcomes.^9,10,11,12,27,28^

## Conclusions

Given the movement toward increasing work requirements, it is critical to examine their association with the receipt of nutrition assistance by low-income adults who are otherwise eligible, particularly for subpopulations that may have greater difficulty meeting requirements. Work requirements are associated with substantial reductions in SNAP participation, including for people with disabilities. Reductions in participation appear to be more severe among non-Hispanic black than non-Hispanic white adults. The likelihood of disparate and unintended harm should be carefully considered in proposals to further expand work requirements.

## Notes

Supplement. **eAppendix.** Supplemental Methods: Analytic Model and Work Requirement Variable **eTable 1.** Characteristics of Analytic Sample (n = 866 000) **eTable 2.** Associations of Work Requirements and SNAP Participation, Difference-in-Difference Models **eTable 3.** Associations of Work Requirements and SNAP Participation: Triple Difference Models Comparing Nondisabled Childless Adults vs Parents **eTable 4.** Associations of Work Requirements and SNAP Participation: Triple Difference Models Comparing Adults Aged 45 to 49 Years vs Those Aged 50 to 54 Years **eTable 5.** Associations of Work Requirements and SNAP Participation for Adults with Disability, Excluding SSI Recipients
