# Supplementary material for: Association of Work Requirements With Supplemental Nutrition Assistance Program Participation by Race/Ethnicity and Disability Status, 2013-2017
Source: JAMA Netw Open. 2020 Jun 26;3(6):e205824. doi: 10.1001/jamanetworkopen.2020.5824 (PMC7320297; doi:10.1001/jamanetworkopen.2020.5824)
Supplement: Supplement. — eAppendix. Supplemental Methods: Analytic Model and Work Requirement Variable eTable 1. Characteristics of Analytic Sample (n = 866 000) eTable 2. Associations of Work Requirements and SNAP Participation, Difference-in-Difference Models eTable 3. Associations of Work Requirements and SNAP Participation: Triple Difference Models Comparing Nondisabled Childless Adults vs Parents eTable 4. Associations of Work Requirements and SNAP Participation: Triple Difference Models Comparing Adults Aged 45 to 49 Years vs Those Aged 50 to 54 Years eTable 5. Associations of Work Requirements and SNAP Participation for Adults with Disability, Excluding SSI Recipients [file jamanetwopen-3-e205824-s001.pdf]

Supplementary Online Content

Brantley E, Pillai D, Ku L. Association of work requirements with Supplemental Nutrition Assistance Program participation by race/ethnicity and disability status, 2013-2017. *JAMA Netw Open.* 2020;3(6):e205824. doi:10.1001/jamanetworkopen.2020.5824

eAppendix. Supplemental Methods: Analytic Model and Work Requirement Variable

eTable 1. Characteristics of Analytic Sample (n = 866,000)

eTable 2. Associations of Work Requirements and SNAP Participation, Difference-in-Difference Models

eTable 3. Associations of Work Requirements and SNAP Participation: Triple Difference Models Comparing Nondisabled Childless Adults vs Parents

eTable 4. Associations of Work Requirements and SNAP Participation: Triple Difference Models Comparing Adults Aged 45 to 49 Years vs Those Aged 50 to 54 Years

eTable 5. Associations of Work Requirements and SNAP Participation for Adults with Disability, Excluding SSI Recipients

This supplementary material has been provided by the authors to give readers additional information about their work.

© 2020 Brantley E et al. *JAMA Network Open*.

eAppendix. Supplemental Methods: Analytic Model and Work Requirement Variable

Analytic models

First, we estimated weighted linear probability models for each sample, using year fixed effects to account for national changes over time and Public Use Microdata Area (PUMA) fixed effects to account for time-invariant associations between PUMA and SNAP participation. This is equivalent to difference-in-difference models:

$$Y_{ipst}=\beta_1 WR_{pt} + \beta_2 X_i + \beta_3 Mcaid\_adult_{st} + \beta_4 UR_{pt} + \beta_5 UR_{p(t-1)} + \beta_6 Pov_{pt} + \alpha_p + \delta_t + \varepsilon_{ipst} \quad \text{(Equation 1)}$$

where *i* indexes individual, *p* indexes Public Use Microdata Area (PUMA), *s* indexes state, and *t* indexes year. β_1 is the coefficient of interest and indicates the association between living in an area with a work requirement and SNAP participation. *WR* is the work requirement variable for each PUMA, described in more detail below. X_i is a vector of individual covariates, comprising age (indicator variables), gender, marital status, education, household size and home ownership (vs. renting). *Mcaid_adult* is the state's Medicaid eligibility for childless adults in the current year, UR_{pt} and $UR_{p(t-1)}$ are PUMA-level unemployment rates in the current and prior years, Pov_{pt} is the current-year poverty rate, α is a set of PUMA fixed effects, δ represents year fixed effects, and ε_{ipst} is the error term.

The PUMA-level unemployment and poverty rates were estimated using ACS data. The unemployment rate is defined as the number of unemployed among all adults (ages 16 or older) in the civilian labor force. The poverty rate is defined as the number of people with incomes below the poverty line among all people.

Next, we estimate triple difference models, using parents who would otherwise meet able-bodied adult without dependents (ABAWD) work requirement criteria as a comparison group for ABAWDs:

$$Y_{ipst}=\beta_1 WR_{pt} + \beta_2 ABAWD_i + \beta_3 WR*ABAWD_{ipt} + \beta_4 X_i + \beta_5 Mcaid\_adult_{st} + \beta_6 Mcaid\_parent_{st} + \beta_7 UR_{pt} + \beta_8 UR_{p(t-1)} + \beta_9 Pov_{pt} + \alpha_p + \delta_t + \varepsilon_{ipst} \quad \text{(Equation 2)}$$

Where ABAWD is an indicator for being an ABAWD (exposed) versus a parent (unexposed). β_3 is the coefficient of interest and indicates the interaction of living in an area with a work requirement with being an ABAWD. This model incorporates parental Medicaid eligibility (*Mcaid_parent*) since parents are included. Other terms are as above. For the disabled sample, we compare disabled parents with disabled childless adults.

As an alternative to comparing parents vs. childless adults, we estimated models comparing childless adults ages 45 to 49 to those ages 50 to 54:

$$Y_{ipst}=\beta_1 WR_{pt} + \beta_2 WR*ABAWD\_age_{ipt} + \beta_3 X_i + \beta_4 Mcaid\_adult_{st} + \beta_5 UR_{pt} + \beta_6 UR_{p(t-1)} + \beta_7 Pov_{pt} + \alpha_p + \delta_t + \varepsilon_{ipst} \quad \text{(Equation 3)}$$

Where *ABAWD_age* is an indicator for the 45 to 49 age group (vs 50 to 54). β_2 is the coefficient of interest representing the interaction of living in a work requirement area with being in the ABAWD age range. Other terms are as in Equation 1. The model does not include a separate term for the 45 to 49 age group since this would be collinear with age indicator variables.

Work requirement variable

© 2020 Brantley E et al. *JAMA Network Open*.

The ACS asks respondents to report any food stamp participation in the past 12 months; the survey is fielded throughout the year. We obtained information from the Food and Nutrition Service on waivers of work requirements for each quarter. We constructed an estimate of each PUMA's exposure to work requirements that reflects the average level of work requirements over the survey year and prior year. We weighted the quarters closer to the middle of the two-year period higher (e.g., fourth quarter of the prior year and first quarter of the index year) because these quarters would be most likely to be included in the 12-month window of a date drawn randomly between January 1 and December 31st. Specifically, we use the following formula to calculate the work requirement variable:

$$WR_{pt} = .05*(Q1_{t-1}) + .1*(Q2_{t-1}) + .15*(Q3_{t-1}) + .2(Q4_{t-1}) + .2*Q1 + .15*Q2 + .1*Q3 + .05*Q4$$

eTable 1: Characteristics of Analytic Sample (n = 866,000)

	mean (%)	95% CI
Race/Ethnicity, %		
non- Hispanic white	56.45	(56.31 to 56.59)
non-Hispanic black	19.39	(19.28 to 19.51)
non-Hispanic Asian	2.54	(2.49 to 2.58)
Hispanic	17.74	(17.64 to 17.85)
Other/multiple race	3.88	(3.83 to 3.93)
Male, %	42.47	(42.33 to 42.6)
Education, %		
Less than high school	15.72	(15.62 to 15.82)
High school	37.67	(37.54 to 37.81)
Some college	33.68	(33.55 to 33.81)
College grad	12.92	(12.83 to 13.01)
Married, %	34.58	(34.45 to 34.7)
Rents, %	66.42	(66.3 to 66.55)
Household size, mean	3.35	(3.34 to 3.35)
Age, mean	33.58	(33.56 to 33.61)
Medicaid eligibility- adults, mean	62.18	(61.99 to 62.36)
Medicaid eligibility- parents, mean	90.84	(90.69 to 90.98)
Unemployment rate, mean	7.34	(7.33 to 7.35)
Poverty rate, mean	17.01	(16.99 to 17.03)

[a.] Weighted estimates of 2013-2017 American Community Survey data.

© 2020 Brantley E et al. *JAMA Network Open*.

eTable 2: Associations of Work Requirements and SNAP Participation, Difference-in-Difference Models[a]

Variable	All ABAWDs	Stratified racial/ethnic subpopulations of ABAWDs: non-Hispanic black	Hispanic	non-Hispanic white	Disabled adults
	Percentage Point Change (95% CI)				
Work requirement	-0.035	-0.06	-0.019	-0.025	-0.044
	(-0.045 to -0.026)	(-0.097 to -0.039)	(-0.047 to 0.009)	(-0.036 to -0.014)	(-0.066 to -0.022)
Race/ethnic group (ref= non-Hispanic white)					
NH black	0.112	n/a	n/a	n/a	0.073
	(0.103 to 0.121)	n/a	n/a	n/a	(0.057 to 0.089)
Asian	-0.041	n/a	n/a	n/a	-0.141
	(-0.050 to -0.031)	n/a	n/a	n/a	(-0.182 to -0.101)
Hispanic	0.004	n/a	n/a	n/a	0.009
	(-0.004 to 0.011)	n/a	n/a	n/a	(-0.010 to 0.029)
Other race	0.062	n/a	n/a	n/a	0.045
	(0.050 to 0.074)	n/a	n/a	n/a	(0.022 to 0.068)
Male	-0.028	-0.051	-0.031	-0.02	-0.053
	(-0.031 to -0.024)	(-0.061 to -0.040)	(-0.042 to -0.021)	(-0.024 to -0.016)	(-0.062 to -0.044)
Educational attainment (ref = no high school)					
High school	-0.085	-0.122	-0.058	-0.089	-0.07
	(-0.093 to -0.078)	(-0.140 to -0.104)	(-0.074 to -0.042)	(-0.099 to -0.078)	(-0.082 to -0.058)
Some college	-0.122	-0.152	-0.065	-0.133	-0.12
	(-0.130 to -0.114)	(-0.172 to -0.132)	(-0.082 to -0.048)	(-0.143 to -0.123)	(-0.133 to -0.107)
College	-0.183	-0.224	-0.119	-0.185	-0.241
	(-0.191 to -0.174)	(-0.247 to -0.200)	(-0.138 to -0.101)	(-0.196 to -0.174)	(-0.260 to -0.222)
Married	-0.033	-0.04	-0.047	-0.026	-0.072
	(-0.040 to -0.027)	(-0.060 to -0.020)	(-0.062 to -0.031)	(-0.033 to -0.018)	(-0.086 to -0.058)
Rents	0.068	0.092	0.078	0.065	0.157
	(0.063 to 0.073)	(0.075 to 0.110)	(0.063 to 0.094)	(0.059 to 0.071)	(0.145 to 0.168)
Household size	0.026	0.036	0.02	0.026	0.016
	(0.023 to 0.029)	(0.028 to 0.045)	(0.014 to 0.027)	(0.023 to 0.030)	(0.010 to 0.022)
Medicaid eligibility	0.011	-0.003	0.013	0.014	0.023
	(0.003 to 0.018)	(-0.026 to 0.020)	(-0.007 to 0.033)	(0.005 to 0.022)	(0.006 to 0.039)
UR rate	0.005	0.005	0.005	0.004	0.004
	(0.003 to 0.006)	(0.001 to 0.009)	(0.001 to 0.010)	(0.002 to 0.006)	(0.001 to 0.007)
UR rate, 1-yr earlier	0.001	0	0.001	0.001	0.002
	(-0.000 to 0.003)	(-0.003 to 0.004)	(-0.003 to 0.004)	(-0.001 to 0.003)	(-0.001 to 0.005)
Poverty rate	0.002	0.003	0.002	0.002	0.002
	(0.001 to 0.003)	(0.000 to 0.006)	(-0.000 to 0.005)	(0.000 to 0.003)	(-0.000 to 0.004)
n	272,397	41,269	35,163	176,140	71,148

Abbreviation: NH=Non-Hispanic UR=Unemployment rate. WR= Work requirement [a] Source: Authors' analysis of 2012-2017 American Community Survey data. Linear probability models with area and year fixed effects. Models also control for age, gender, marital status, education, household size and home ownership.

© 2020 Brantley E et al. *JAMA Network Open*.

eTable 3: Associations of Work Requirements and SNAP Participation, Triple Difference Models Comparing Nondisabled Childless Adults vs Parents [a]

		Stratified racial/ethnic subpopulations of ABAWDs			
	All ABAWDs	non-Hispanic black	Hispanic	non-Hispanic white	Disabled adults
Variable	Percentage Point Change (95% CI)				
Work requirement	-0.006 (-0.014 to 0.002)	-0.002 (-0.021 to 0.016)	-0.005 (-0.024 to 0.013)	-0.006 (-0.016 to 0.004)	-0.009 (-0.027 to 0.010)
Childless adults (vs parents)	-0.178 (-0.184 to -0.172)	-0.175 (-0.189 to -0.162)	-0.181 (-0.194 to -0.168)	-0.171 (-0.179 to -0.164)	-0.080 (-0.092 to -0.069)
WR*Childless interaction	-0.040 (-0.048 to -0.032)	-0.072 (-0.092 to -0.051)	-0.055 (-0.072 to -0.038)	-0.026 (-0.035 to -0.016)	-0.040 (-0.058 to -0.023)
Race/ethnic group (ref= non-Hispanic white)					
NH black	0.114 (0.108 to 0.120)	n/a n/a	n/a n/a	n/a n/a	0.063 (0.052 to 0.074)
Asian	-0.031 (-0.040 to -0.022)	n/a n/a	n/a n/a	n/a n/a	-0.12 (-0.152 to -0.087)
Hispanic	0.009 (0.003 to 0.015)	n/a n/a	n/a n/a	n/a n/a	0.011 (-0.002 to 0.024)
Other race	0.067 (0.058 to 0.075)	n/a n/a	n/a n/a	n/a n/a	0.049 (0.033 to 0.065)
Male	-0.053 (-0.055 to -0.050)	-0.085 (-0.092 to -0.077)	-0.061 (-0.067 to -0.056)	-0.04 (-0.043 to -0.038)	-0.069 (-0.076 to -0.063)
Educational attainment (ref = no high school)					
High school	-0.089 (-0.094 to -0.084)	-0.118 (-0.129 to -0.108)	-0.069 (-0.078 to -0.060)	-0.1 (-0.107 to -0.092)	-0.079 (-0.087 to -0.070)
Some college	-0.132 (-0.137 to -0.127)	-0.156 (-0.168 to -0.145)	-0.085 (-0.095 to -0.076)	-0.152 (-0.160 to -0.145)	-0.117 (-0.126 to -0.108)
College	-0.224 (-0.230 to -0.218)	-0.258 (-0.273 to -0.243)	-0.159 (-0.172 to -0.147)	-0.243 (-0.251 to -0.235)	-0.256 (-0.271 to -0.242)
Married	-0.12 (-0.124 to -0.115)	-0.122 (-0.132 to -0.111)	-0.124 (-0.132 to -0.115)	-0.111 (-0.116 to -0.106)	-0.103 (-0.112 to -0.094)
Rents	0.15 (0.146 to 0.154)	0.157 (0.146 to 0.168)	0.164 (0.156 to 0.172)	0.145 (0.140 to 0.150)	0.186 (0.178 to 0.194)
Household size	0.034 (0.033 to 0.036)	0.042 (0.039 to 0.046)	0.041 (0.038 to 0.044)	0.029 (0.027 to 0.031)	0.02 (0.017 to 0.024)
Mcaid elig.- adults	0.01 (0.003 to 0.017)	-0.003 (-0.022 to 0.015)	0.016 (-0.001 to 0.034)	0.01 (0.001 to 0.018)	0.016 (-0.001 to 0.032)
Mcaid elig.- parents	-0.01 (-0.020 to 0.001)	0.004 (-0.024 to 0.033)	-0.002 (-0.035 to 0.032)	-0.013 (-0.025 to -0.001)	-0.01 (-0.020 to 0.001)
UR rate	0.004 (0.003 to 0.005)	0.004 (0.002 to 0.006)	0.002 (-0.001 to 0.004)	0.005 (0.003 to 0.006)	0.003 (0.001 to 0.005)
UR rate, 1-yr earlier	0.001 (0.000 to 0.002)	0 (-0.002 to 0.002)	0.001 (-0.001 to 0.003)	0.002 (0.000 to 0.003)	0 (-0.002 to 0.003)
Poverty rate	0.004 (0.003 to 0.004)	0.003 (0.001 to 0.005)	0.004 (0.002 to 0.005)	0.004 (0.003 to 0.005)	0.004 (0.003 to 0.006)
n	735,302	109,330	121,189	451,503	130,698

Abbreviation: NH = Non-Hispanic. UR=Unemployment rate. WR= Work requirement [a] Source: Authors' analysis of 2012-2017 American Community Survey data. Linear probability models with area and year fixed effects. Models also control for age, gender, marital status, education, household size and home ownership.

eTable 4: Associations of Work Requirements and SNAP Participation, Triple Difference Models Comparing Adults Aged 45 to 49 Years vs Those Aged 50 to 54 Years

Variable	All ABAWDs	Stratified racial/ethnic subpopulations of ABAWDs: non-Hispanic black	Hispanic	non-Hispanic white	Disabled adults
	Percentage Point Change (95% CI)				
Work requirement	-0.026 (-0.041 to -0.010)	-0.031 -0.076 to 0.014)	-0.02 (-0.078 to 0.038)	-0.029 (-0.049 to -0.010)	0.007 (-0.018 to 0.033)
WR* 45 to 49 interaction	-0.021 (-0.036 to -0.007)	-0.059 (-0.101 to -0.018)	-0.003 (-0.045 to 0.039)	-0.012 (-0.030 to 0.005)	-0.04 (-0.065 to -0.016)
Race/ethnic group (ref= non-Hispanic white)					
NH black	0.076 (0.064 to 0.087)	n/a n/a	n/a n/a	n/a n/a	0.065 (0.050 to 0.081)
Asian	-0.063 (-0.078 to -0.047)	n/a n/a	n/a n/a	n/a n/a	-0.114 (-0.169 to -0.059)
Hispanic	-0.022 (-0.035 to -0.010)	n/a n/a	n/a n/a	n/a n/a	0.021 (-0.000 to 0.043)
Other race	0.031 (0.010 to 0.051)	n/a n/a	n/a n/a	n/a n/a	0.056 (0.030 to 0.082)
Male	-0.023 (-0.028 to -0.017)	-0.038 (-0.054 to -0.022)	-0.026 (-0.044 to -0.008)	-0.013 (-0.020 to -0.006)	-0.068 (-0.077 to -0.059)
Educational attainment (ref = no high school)					
High school	-0.079 (-0.089 to -0.069)	-0.12 (-0.145 to -0.095)	-0.042 (-0.069 to -0.015)	-0.082 (-0.096 to -0.069)	-0.078 (-0.090 to -0.065)
Some college	-0.098 (-0.109 to -0.088)	-0.142 (-0.169 to -0.116)	-0.042 (-0.073 to -0.012)	-0.104 (-0.118 to -0.090)	-0.089 (-0.102 to -0.075)
College	-0.147 (-0.159 to -0.136)	-0.182 (-0.217 to -0.147)	-0.087 (-0.124 to -0.049)	-0.154 (-0.170 to -0.139)	-0.194 (-0.215 to -0.173)
Married	-0.069 (-0.077 to -0.060)	-0.086 (-0.113 to -0.060)	-0.071 (-0.095 to -0.047)	-0.065 (-0.074 to -0.055)	-0.115 (-0.129 to -0.101)
Rents	0.119 (0.112 to 0.127)	0.127 (0.108 to 0.147)	0.103 (0.079 to 0.126)	0.122 (0.113 to 0.131)	0.185 (0.173 to 0.196)
Household size	0.034 (0.029 to 0.038)	0.042 (0.031 to 0.054)	0.017 (0.006 to 0.029)	0.039 (0.033 to 0.045)	0.03 (0.023 to 0.036)
Medicaid eligibility	0.016 (0.005 to 0.026)	-0.017 (-0.047 to 0.014)	0.024 (-0.014 to 0.063)	0.024 (0.011 to 0.036)	0.035 (0.019 to 0.052)
UR rate	0.003 (0.001 to 0.005)	0 (-0.004 to 0.005)	0.001 (-0.007 to 0.008)	0.004 (0.001 to 0.007)	-0.001 (-0.004 to 0.003)
UR rate, 1-yr earlier	0.002 (0.000 to 0.004)	0.004 (-0.000 to 0.008)	0 (-0.007 to 0.006)	0.001 (-0.001 to 0.004)	0.001 (-0.002 to 0.004)
Poverty rate	0.002 (0.000 to 0.003)	0.006 (0.002 to 0.009)	0.001 (-0.004 to 0.006)	0.001 (-0.001 to 0.002)	0.002 (-0.001 to 0.004)
n	109,933	18,454	11,158	72,981	65,847

Abbreviation: NH= Non-Hispanic UR=Unemployment rate. WR= Work requirement [a] Source: Authors' analysis of 2012-2017 American Community Survey data. Linear probability models with area and year fixed effects. Models also control for age, gender, marital status, education, household size and home ownership.

© 2020 Brantley E et al. *JAMA Network Open*.

eTable 5: Associations of Work Requirements and SNAP Participation for Adults with Disability, Excluding SSI Recipients [a]

Variable	Difference in Difference	Triple difference method 1[b]	Triple difference method 2[c]
	Percentage Point Change (95% CI)		
Work requirement	-0.052<br>(-0.078 to -0.027)	0<br>(-0.021 to 0.020)	-0.006<br>(-0.037 to 0.024)
Childless adults (vs parents)	n/a<br>n/a	-0.091<br>(-0.104 to -0.079)	n/a<br>n/a
WR* comparison interaction	n/a<br>n/a	-0.063<br>(-0.082 to -0.044)	-0.036<br>(-0.065 to -0.008)
Race/ethnic group (ref= non-Hispanic white)			
NH black	0.081<br>(0.062 to 0.100)	0.068<br>(0.054 to 0.082)	0.068<br>(0.049 to 0.087)
Asian	-0.144<br>(-0.186 to -0.101)	-0.124<br>(-0.158 to -0.090)	-0.141<br>(-0.203 to -0.078)
Hispanic	-0.001<br>(-0.023 to 0.022)	0.002<br>(-0.013 to 0.017)	0.004<br>(-0.023 to 0.030)
Other race	0.05<br>(0.024 to 0.076)	0.055<br>(0.036 to 0.073)	0.046<br>(0.016 to 0.076)
Male	-0.042<br>(-0.053 to -0.031)	-0.063<br>(-0.071 to -0.055)	-0.052<br>(-0.064 to -0.041)
Educational attainment (ref = no high school)			
High school	-0.075<br>(-0.090 to -0.060)	-0.082<br>(-0.092 to -0.072)	-0.067<br>(-0.082 to -0.051)
Some college	-0.114<br>(-0.130 to -0.099)	-0.115<br>(-0.125 to -0.104)	-0.08<br>(-0.096 to -0.063)
College	-0.239<br>(-0.260 to -0.218)	-0.257<br>(-0.272 to -0.241)	-0.171<br>(-0.195 to -0.147)
Married	-0.074<br>(-0.090 to -0.058)	-0.111<br>(-0.120 to -0.101)	-0.122<br>(-0.137 to -0.106)
Rents	0.146<br>(0.133 to 0.159)	0.179<br>(0.170 to 0.188)	0.175<br>(0.162 to 0.188)
Household size	0.026<br>(0.019 to 0.033)	0.026<br>(0.022 to 0.029)	0.044<br>(0.036 to 0.052)
Mcaid elig.-adults	0.027<br>(0.008 to 0.046)	0.015<br>(-0.004 to 0.033)	0.04<br>(0.021 to 0.060)
Mcaid elig.-parents	n/a<br>n/a	-0.013<br>(-0.038 to 0.013)	n/a<br>n/a
UR rate	0.004<br>(-0.000 to 0.007)	0.003<br>(0.000 to 0.005)	-0.002<br>(-0.006 to 0.002)
UR rate, 1-yr earlier	0.004<br>(0.000 to 0.007)	0.002<br>(-0.001 to 0.004)	0.002<br>(-0.001 to 0.006)
Poverty rate	0.002<br>(-0.000 to 0.005)	0.005<br>(0.003 to 0.007)	0.002<br>(-0.001 to 0.005)
n	51,123	100,277	46,764

Abbreviation: NH= Non-Hispanic UR=Unemployment rate. WR= Work requirement [a] Source: Authors' analysis of 2012-2017 American Community Survey data. Linear probability models with area and year fixed effects. Models also control for age, gender, marital status, education, household size and home ownership.
[b] Comparing childless adults to parents. [c] Comparing adults ages 45 to 49 to those ages 50 to 54.

© 2020 Brantley E et al. *JAMA Network Open*.
